# Supplementary material for: Entangled Interlocked Diamond-like (Diamondiynes) Lattices
Source: ACS Omega. 2025 Sep 25;10(39):46065–70. doi: 10.1021/acsomega.5c07159 (PMC12508917; doi:10.1021/acsomega.5c07159)
Supplement: Supplementary file 1 [file ao5c07159_si_001.pdf]

Supporting Information: Entangled Interlocked Diamond-like (Diamondiynes) Lattices

C. M. O. Bastos,[†] E. J. A. dos Santos,[‡] R. A. F. Alves,[‡] A. C. Dias,[†] L. A. R. Junior,[‡] and D. S. Galvão[¶]

†*Institute of Physics and International Center of Physics, University of Brasília, Brasília* 70919-970, *DF, Brazil*

‡*Computational Materials Laboratory, LCCMat, Institute of Physics, University of Brasília, 70910-900, Brasília, Federal District, Brazil.*

¶*Department of Applied Physics and Center for Computational Engineering and Sciences, State University of Campinas, Campinas, 13083-859, SP, Brazil*

E-mail:

S1 Optimized fdf structural files

S1.1 NI

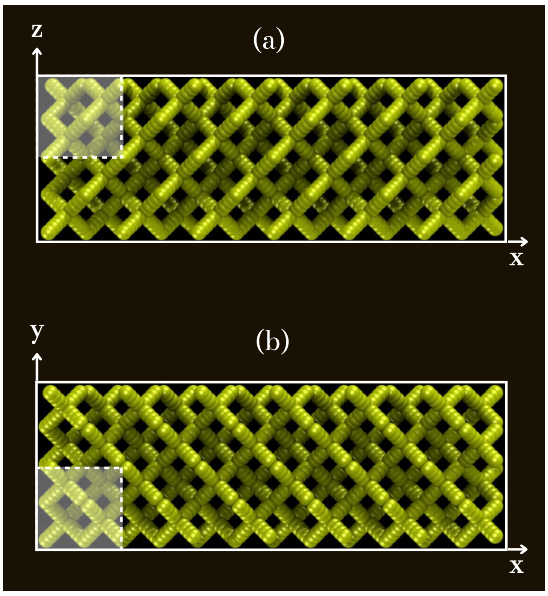

Figure S1: Atomic structure of the NI phase, characterized by a single interwoven sublattice highlighted in yellow. The projections onto the xz (a) and xy (b) planes reveal the regular interlocking pattern. The primitive unit cell is indicated by the shaded region in both panels

Listing 1: NI phase fdf structural file

```
%block ChemicalSpeciesLabel
  1   6   C
%endblock ChemicalSpeciesLabel

LatticeConstant   1.0   Ang
AtomicCoordinatesFormat   Fractional

%block LatticeVectors
-7.840844126       -7.840844114        0.012439886
-7.840844112        0.012439872       -7.840844110
0.012439884       -7.840844111       -7.840844122
%endblock LatticeVectors


%block AtomicCoordinatesAndAtomicSpecies
```

```
0.749804426  0.749804426  0.749804422  1
0.000196087  0.000196087  0.000196083  1
0.804238362  0.586495149  0.804238364  1
0.945761391  0.945761391  0.163505035  1
0.586495149  0.804238363  0.804238364  1
0.945761390  0.163505036  0.945761390  1
0.804353677  0.804353677  0.804353676  1
0.945646966  0.945646968  0.945646968  1
0.804238364  0.804238363  0.586495148  1
0.163505037  0.945761388  0.945761391  1
0.450213162  0.849848065  0.849848066  1
0.299786939  0.900150275  0.900150276  1
0.849848064  0.450213162  0.849848064  1
0.900123092  0.900123092  0.900123092  1
0.849848065  0.849848065  0.450213161  1
0.900150274  0.299786939  0.900150274  1
0.849877594  0.849877596  0.849877596  1
0.900150275  0.900150274  0.299786938  1
%endblock AtomicCoordinatesAndAtomicSpecies
```

S1.2 2f-Symmetry

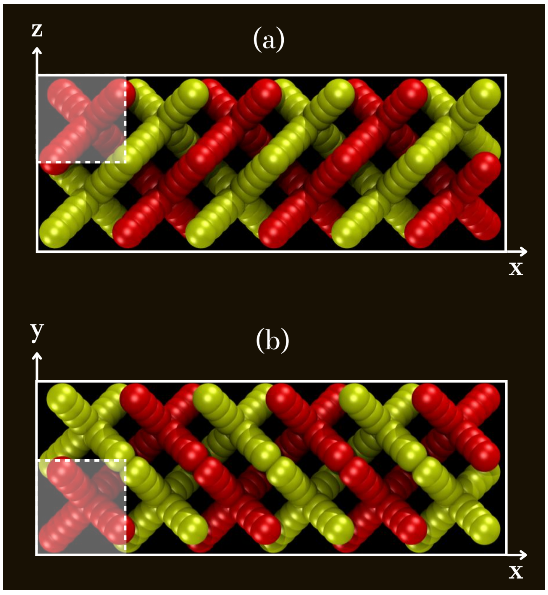

Figure S2: Atomic structure of the 2f-sym phase, consisting of interpenetrated sublattices highlighted in red and yellow. The panels display projections onto different crystallographic planes: (a) projection onto the xz plane; and (b) projection onto the xy plane. The images highlight the regular and symmetric interlocking between sublattices, with the primitive unit cell indicated by the shaded region in each panel.

Listing 2: 2f-symmetry phase fdf structural file

```
%block ChemicalSpeciesLabel
  1   6   C
%endblock ChemicalSpeciesLabel

LatticeConstant   1.0   Ang
AtomicCoordinatesFormat   Fractional

%block LatticeVectors
7.846924598       -0.000000002       0.000000001
-0.000000002        7.846924593      -0.000000000
0.000000001       -0.000000000       7.846924597
%endblock LatticeVectors


%block AtomicCoordinatesAndAtomicSpecies
0.500000000   0.500000000   0.500000000   1
```

```
-0.000000000  0.000000000  -0.000000000  1
0.608919528  0.391080471  0.608919529  1
0.108919528  0.891080472  0.891080471  1
0.608919529  0.608919529  0.391080471  1
0.891080471  0.108919529  0.891080471  1
0.391080471  0.391080471  0.391080471  1
0.108919529  0.108919529  0.108919529  1
0.391080471  0.608919529  0.608919529  1
0.891080470  0.891080471  0.108919530  1
0.699825577  0.699825577  0.300174423  1
0.800174423  0.800174423  0.199825577  1
0.699825579  0.300174422  0.699825578  1
0.199825577  0.199825577  0.199825577  1
0.300174423  0.699825577  0.699825577  1
0.800174422  0.199825578  0.800174422  1
0.300174423  0.300174422  0.300174423  1
0.199825579  0.800174422  0.800174422  1
%endblock AtomicCoordinatesAndAtomicSpecies
```

S1.3 2f-Unsymmetry

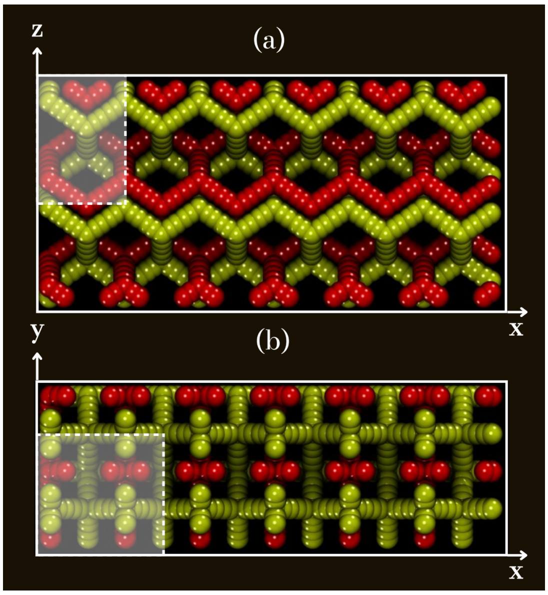

Figure S3: Atomic structure of the 2f-unsym phase, consisting of interpenetrated sublattices highlighted in red and yellow. The panels display projections onto different crystallographic planes: (a) projection onto the xz plane; and (b) projection onto the xy plane. The images highlight the interlocking between sublattices, with the primitive unit cell indicated by the shaded region in each panel.

Listing 3: 2f-unsymmetry phase fdf structural file

```
 %block ChemicalSpeciesLabel
   1   6   C
 %endblock ChemicalSpeciesLabel

 LatticeConstant  1.0  Ang
 AtomicCoordinatesFormat  Fractional

 %block LatticeVectors
4.924299092       4.924042334       9.224090784
4.963672650      -4.922204775      -9.253232241
-4.922461533       4.963693790      -9.253428750
 %endblock LatticeVectors


 %block AtomicCoordinatesAndAtomicSpecies
 0.498110674  0.885540461  0.388053092  1
```

```
-0.001887079  0.138059770  0.135557839  1
0.001875072  0.864456053  0.861959199  1
0.501868320  0.611951432  0.114447646  1
0.377852717  0.933649756  0.315978132  1
0.117627571  0.305591179  0.183396830  1
0.882364175  0.816609608  0.694411135  1
0.382367869  0.444408678  0.066611359  1
0.617624010  0.933392692  0.555599415  1
0.622128791  0.684027938  0.066357714  1
0.122129166  0.816360928  0.934033280  1
0.877851173  0.065979477  0.183645619  1
0.702284342  0.983630373  0.688849109  1
0.793887226  0.030461801  0.233944852  1
0.206100574  0.766072458  0.969526732  1
0.706100227  0.719543184  0.016064842  1
0.293885199  0.983942179  0.280477519  1
0.297704833  0.311158285  0.016370703  1
0.797707791  0.766372868  0.561159091  1
0.202287010  0.438847495  0.233631168  1
0.379350902  0.718372164  0.339589563  1
0.879360163  0.089595742  0.968367660  1
0.120632007  0.031621184  0.910422787  1
0.620632684  0.660412082  0.281632296  1
0.382268085  0.660440164  0.043360938  1
0.882276803  0.793365656  0.910456122  1
0.617708390  0.956631690  0.339554548  1
0.117709466  0.089560457  0.206643791  1
0.700049513  0.989631451  0.288544121  1
0.200049781  0.038538974  0.239650151  1
0.799934550  0.760369865  0.961472968  1
0.299937504  0.711473716  0.010351925  1
0.703422026  0.711791629  0.413790141  1
0.203416350  0.163787898  0.961794295  1
0.296575609  0.586218332  0.288207621  1
0.796575857  0.038209984  0.836215201  1
```

```
%endblock AtomicCoordinatesAndAtomicSpecies
```

S1.4 3f

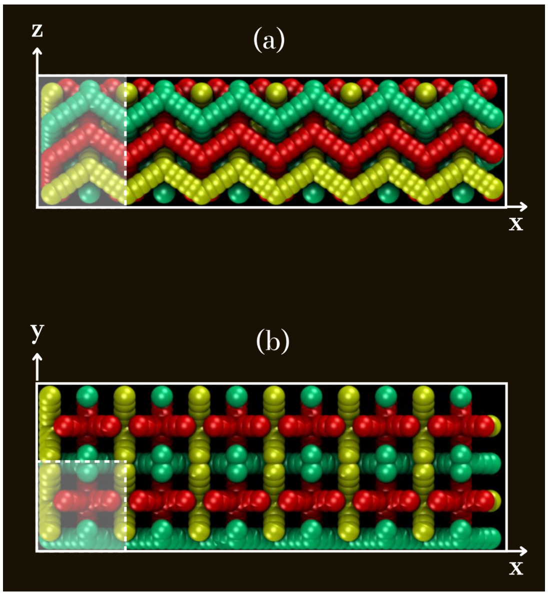

Figure S4: Atomic structure of the 3F phase, consisting of three interpenetrated sublattices highlighted in yellow, red, and green. The panels show projections onto the xz (a) and xy (b) planes, revealing the complex interlocking pattern. The primitive unit cell is indicated by the shaded region.

Listing 4: 3f phase fdf structural file

```
%block ChemicalSpeciesLabel
  1   6   C
%endblock ChemicalSpeciesLabel

LatticeConstant  1.0  Ang
AtomicCoordinatesFormat  Fractional

%block LatticeVectors
-0.000096821       0.000002823      -3.611100927
-6.191959346      -6.189151307      -1.805650173
-6.191957217       6.189152001      -1.805656161
%endblock LatticeVectors

```

```
%block AtomicCoordinatesAndAtomicSpecies
-0.000198575  -0.000054463  -0.000054316  1
0.750844881  0.499963861  0.499963523  1
0.219111916  0.898277079  0.101578748  1
0.868538338  0.601576303  0.601575844  1
0.531536035  0.398322736  0.601621887  1
0.882096026  0.898337309  0.898337739  1
0.219112080  0.101578648  0.898277063  1
0.679087316  0.101598605  0.101598784  1
0.531536393  0.601622218  0.398322433  1
0.071554869  0.398311896  0.398311375  1
0.308199587  0.198702447  0.801186280  1
0.257245979  0.301208338  0.301208293  1
0.442493624  0.698720060  0.301198276  1
0.493464522  0.198708132  0.198708188  1
0.308199593  0.801186432  0.198702594  1
0.890949622  0.801205164  0.801205309  1
0.442493369  0.301198328  0.698719755  1
0.859761078  0.698702748  0.698702857  1
%endblock AtomicCoordinatesAndAtomicSpecies
```

S1.5 4f

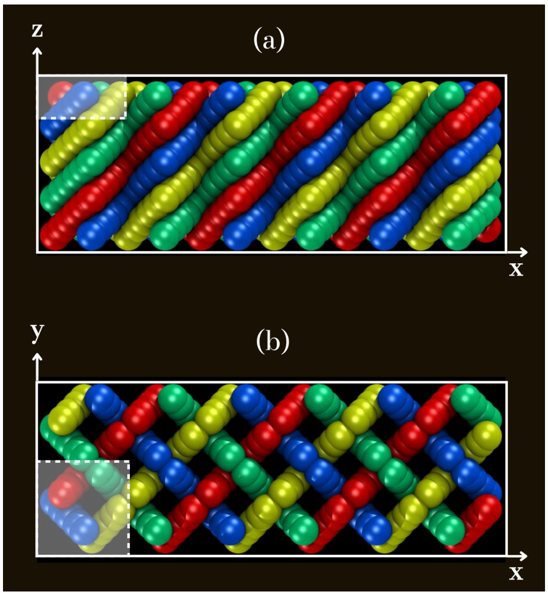

Figure S5: Atomic structure of the 4F phase, composed of four interwoven sublattices highlighted in red, yellow, green, and blue. The projections onto the xz (a) and xy (b) planes reveal the complex and highly organized interlocking pattern. The primitive unit cell is indicated by the shaded region.

Listing 5: 4f phase fdf structural file

```
 %block ChemicalSpeciesLabel
   1   6   C
 %endblock ChemicalSpeciesLabel

 LatticeConstant  1.0  Ang
 AtomicCoordinatesFormat  Fractional

 %block LatticeVectors
6.563174993       0.000012818       0.000012737
0.000012818       6.563204555      -0.000015781
0.000006369      -0.000007892       4.935185919
 %endblock LatticeVectors


 %block AtomicCoordinatesAndAtomicSpecies
 0.499998254  0.000000806  -0.000000964  1
 0.000000699  0.499998445  -0.000002458  1
```

```
0.371993893  0.871997596  0.183958078  1
0.128004989  0.371994772  0.183956657  1
0.628003444  0.871996391  0.816040580  1
0.371993653  0.128004445  0.816040874  1
0.871995571  0.628002761  0.183957073  1
0.128005519  0.628002329  0.816039924  1
0.871996258  0.371994842  0.816039233  1
0.628002359  0.128004577  0.183957716  1
0.706568415  0.206569081  0.388148367  1
0.793430471  0.706568268  0.388148968  1
0.293429293  0.206569298  0.611849550  1
0.706568890  0.793430583  0.611849514  1
0.206568694  0.293430699  0.388149067  1
0.793429859  0.293430515  0.611848664  1
0.206569547  0.706568870  0.611849242  1
0.293429981  0.793431107  0.388148815  1
%endblock AtomicCoordinatesAndAtomicSpecies
```

S2 Electronic Structure

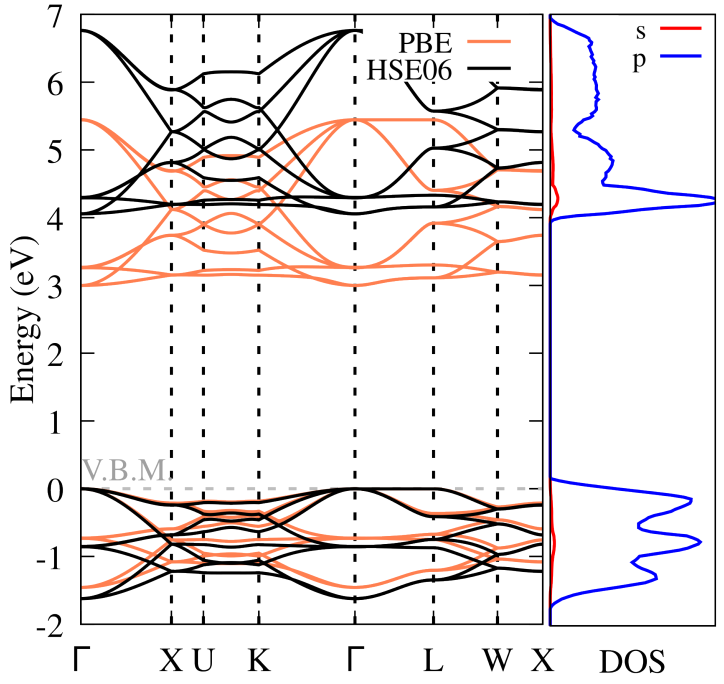

Figure S6: Band structure for non-interpenetrated diamondiyne crystal using PBE and HSE06 exchange correlation functional. The density of state was calculated using HSE06. The zero energy was set in the valence band maximum (VBM)

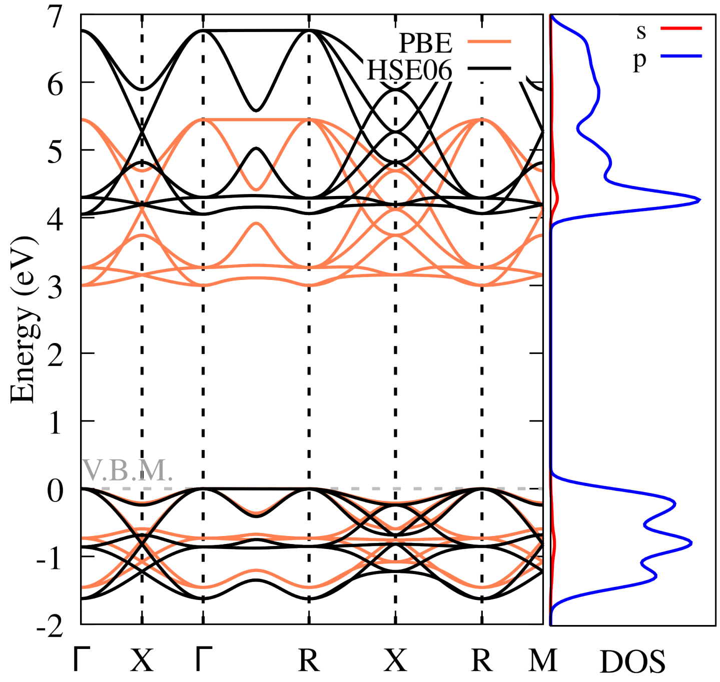

Figure S7: Band structure for symmetric two-fold interpenetrated diamondiyne crystal using PBE and HSE06 exchange correlation functional. The density of state was calculated using HSE06. The zero energy was set in the valence band maximum (VBM)

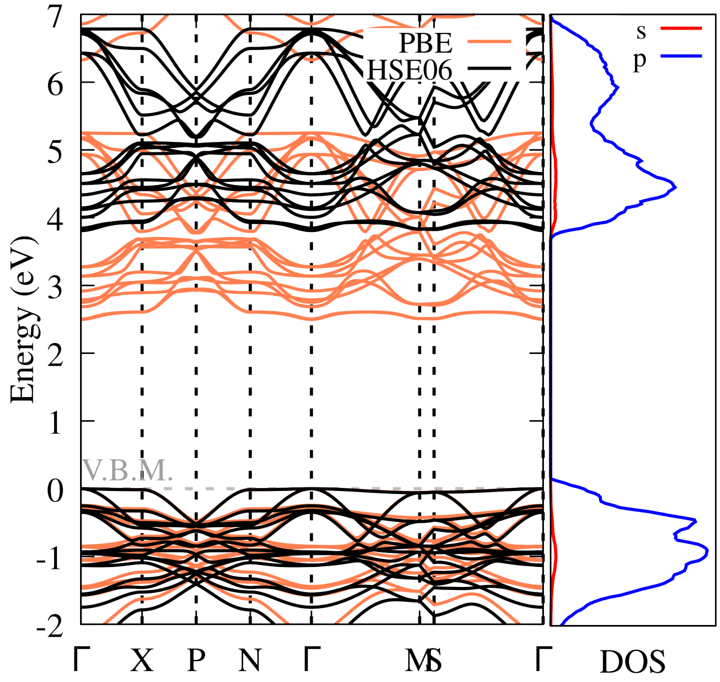

Figure S8: Band structure for unsymmetric two-fold interpenetrated diamondiyne crystal using PBE and HSE06 exchange correlation functional. The density of state was calculated using HSE06. The zero energy was set in the valence band maximum (VBM)

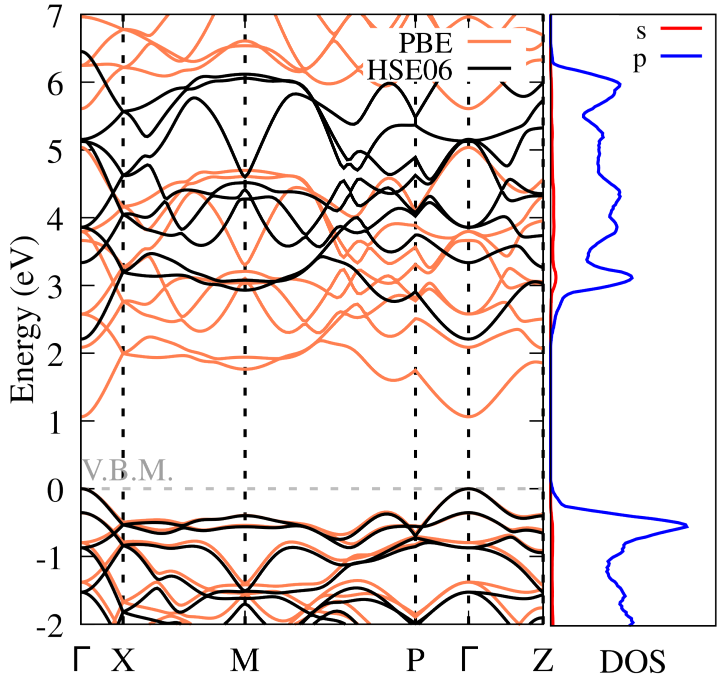

Figure S9: Band structure for three-fold interpenetrated diamondiyne crystal using PBE and HSE06 exchange correlation functional. The density of state was calculated using HSE06. The zero energy was set in the valence band maximum (VBM)

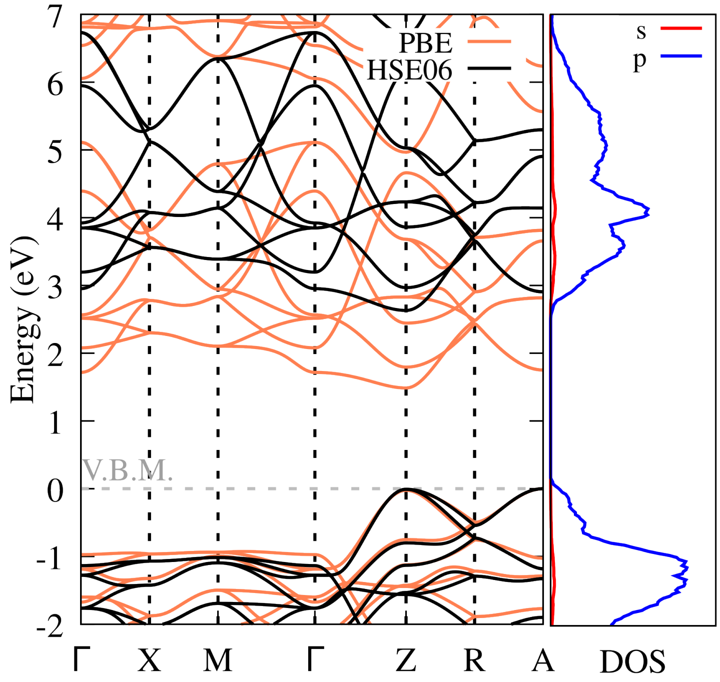

Figure S10: Band structure for four-fold interpenetrated diamondiyne crystal using PBE and HSE06 exchange correlation functional. The density of state was calculated using HSE06. The zero energy was set in the valence band maximum (VBM)

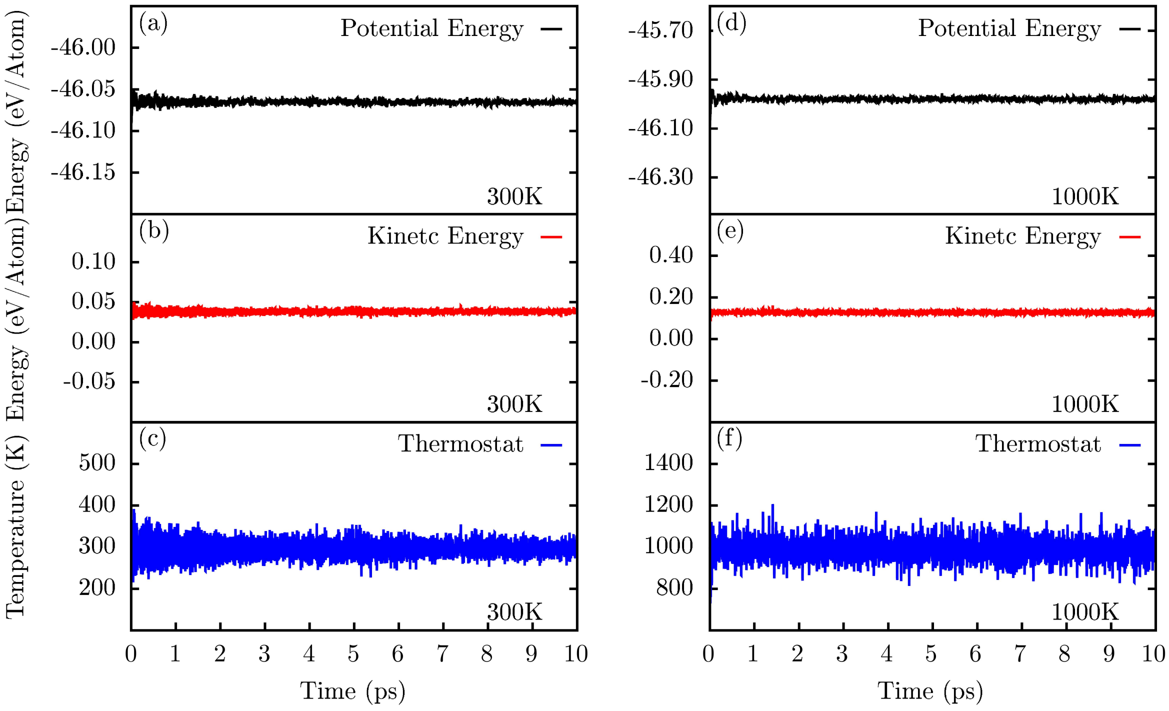

Figure S11: Ab initio molecular dynamics energies as a function of time, showing potential energies at (a) 300 K and (d) 1000 K, kinetic energies at (b) 300 K and (e) 1000 K. The thermostats are shown in panels (c) for 300 K and (f) for 1000 K.

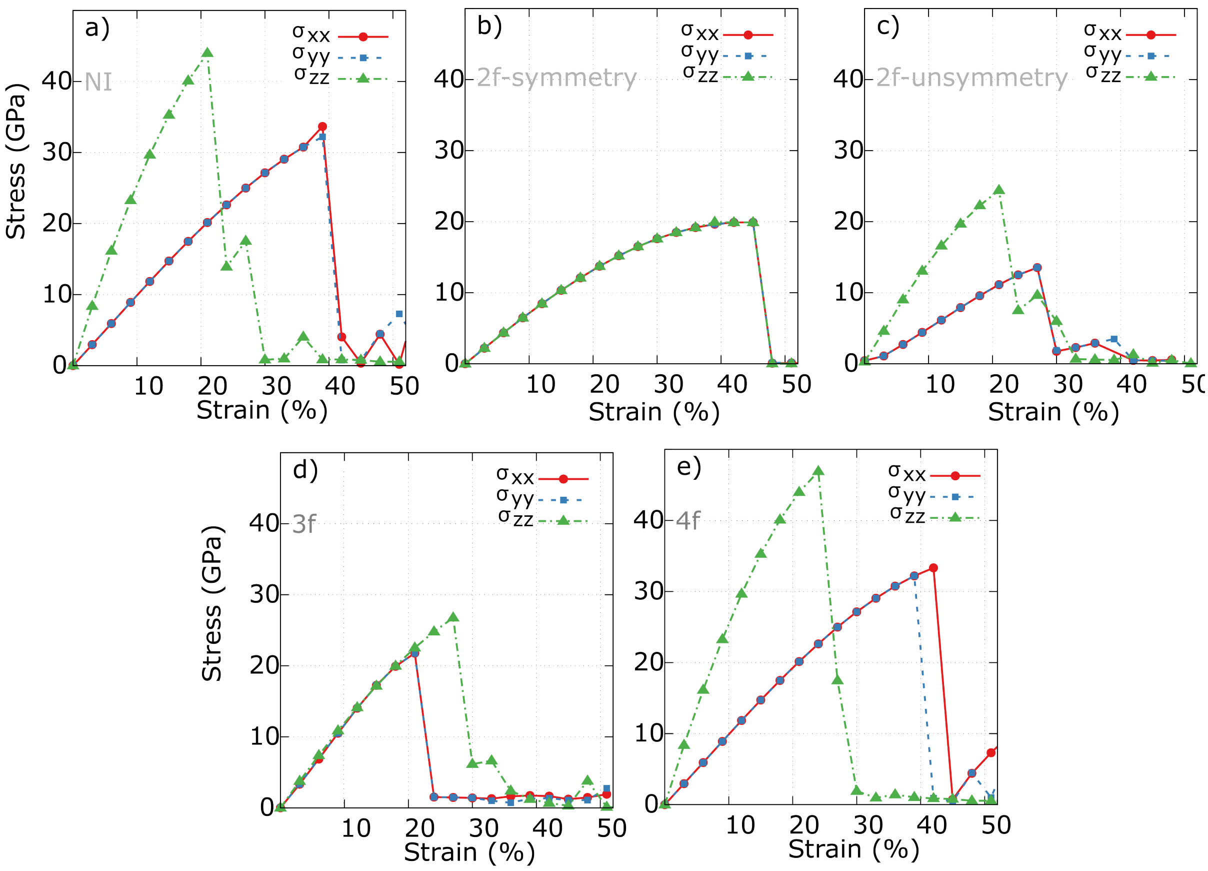

Figure S12: Stress-strain curves under uniaxial deformation along the x (solid red lines with circles), y (dashed blue lines with squares), and z (dash-dotted green lines with triangles) directions for the NI, 2f-symmetry, 2f-unsymmetry, 3f, and 4f phases. The plots highlight the anisotropic behavior of the structures and their distinct mechanical strength limits along each direction.
